# Research Advances in Tissue Engineering Materials for Sustained Release of Growth Factors

**DOI:** 10.1155/2015/808202

**Published:** 2015-08-11

**Authors:** Hai-yang Zhao, Jiang Wu, Jing-jing Zhu, Ze-cong Xiao, Chao-chao He, Hong-xue Shi, Xiao-kun Li, Shu-lin Yang, Jian Xiao

**Affiliations:** ^1^School of Environmental and Biological Engineering, Nanjing University of Science and Technology, Nanjing, Jiangsu 210094, China; ^2^Molecular Pharmacology Research Center, Key Laboratory of Biotechnology and Pharmaceutical Engineering, School of Pharmaceutical Science, Wenzhou Medical University, Wenzhou, Zhejiang 325035, China

## Abstract

Growth factors are a class of cytokines that stimulate cell growth and are widely used in clinical practice, such as wound healing, revascularization, bone repair, and nervous system disease. However, free growth factors have a short half-life and are instable *in vivo*. Therefore, the search of excellent carriers to enhance sustained release of growth factors *in vivo* has become an area of intense research interest. The development of controlled-release systems that protect the recombinant growth factors from enzymatic degradation and provide sustained delivery at the injury site during healing should enhance the growth factor's application in tissue regeneration. Thus, this study reviews current research on commonly used carriers for sustained release of growth factors and their sustained release effects for preservation of their bioactivity and their accomplishment in tissue engineering approaches.

## 1. Introduction

The structure and mechanism of growth factors have been an area of intense research interest as a number of growth factors such as nerve growth factor (NGF) [1], epidermal growth factor (EGF) [2], fibroblast growth factor (FGF) [3], and platelet-derived growth factor (PDGF) [4] have been discovered since the 1950s.

Growth factors are a large class of cytokines that stimulate cell growth and are capable of specifically binding cell membrane receptors seen Table 1. They regulate cell growth, proliferation, migration, and other cellular functions and play important roles in wound healing [5], tissue regeneration [6], and immune regulation [7]. Growth factors cover a broad spectrum of cytokines, including EGFs, NGFs [8], insulin-like growth factors (IGF) [9], FGFs, PDGFs, interleukins, and hematopoietic cell growth factors.

The study of growth factors has long moved to the molecular level after half a century of research. Some growth factors have now been made into preparations that are used clinically with significant effects. However, growth factors have a short half-life and are prone to burst in the body, often making it difficult to reach ideal drug concentrations. Therefore, how to extend the action time of growth factors in the body and maintain proper drug concentrations has become a new area of research.

Ideal carriers for sustained release of growth factors should meet the following criteria. First, carriers have high drug loading and can maintain sustained release of growth factors to ensure reasonable treatment time and efficacy. Second, the addition of carriers does not undermine the biological activity of growth factors. Third, carriers are biocompatible and their residues or degradation products are not cytotoxic. Numerous materials are currently available as sustained release carriers of growth factors seen in Table 2, which can be divided into organic materials and inorganic materials. Organic materials include collagen, gelatin, hyaluronic acid, chitosan, poly(ethylene argininylaspartate diglyceride) (PEAD) [10], poly-L-lactide (PLLA) [11], and poly-lactic-co-glycolic acid (PLGA) [12]. Inorganic materials include calcium phosphate and hydroxyapatite. Organic materials can also be used in combination with inorganic materials, such as porous hydroxyapatite/collagen composite. This study reviews the characteristics and applications of common sustained release carriers for growth factors in an attempt to provide reference for related research and clinical application.

## 2. Organic Materials

Organic materials can be divided into natural materials and composite materials according to their sources. Organic materials can be made into sustained release systems in different formulations such as microspheres and nanoparticles according to their different properties. Currently available natural materials, such as gelatin, alginate, and chitosan, are natural polymers, which can associate with growth factors to function as sustained release carriers* in vivo*. Composite materials include widely used PEAD [10], PLLA [11], and PLGA [12].

### 2.1. Natural Materials

Natural materials offer many advantages as sustained release carriers for growth factors, such as reducing rejection and immune stress and having good biodegradability. For example, natural materials such as collagen, hyaluronic acid, and fibrin are naturally occurring in the body, with collagen accounting for nearly one quarter of human proteins by weight. Natural materials from other sources, such as alginate and chitosan, also have many advantages. They can minimize toxicity and chronic inflammation. In addition, they are also highly biocompatible [13], less irritant to the body, and easy to be degraded.

#### 2.1.1. Gelatin

Gelatin is a natural material produced from hydrolysis of collagen at higher temperatures and is often used as a sustained release carrier for growth factors. Depending on the types of gelatin used, gelatin particles may be positively charged or negatively charged [14]. Growth factors can be efficiently encapsulated in the gelatin particles. This strategy does not compromise the biological activity of growth factors and provides sustained release of growth factors by controlling the release rate of growth factors from gelatin particles.

Hori et al. [15] designed a sustained release system for ophthalmic application of EGF using cationized gelatin hydrogel (CGH) as the carrier. They placed CGH with incorporated ^125^I-labelled EGF in the conjunctival sac of mice and measured the residual radioactivity at different times to evaluate EGF release. The results showed that about 60–67% of EGF applied remained one day after application and about 10–12% of EGF remained seven days after application. Compared with the topical application of EGF solution or blank CGH membranes alone, CGH membranes with incorporated EGF can reduce corneal epithelial defect and promote significant proliferation of epithelial cells, thereby accelerating ocular wound healing. Oe et al. [16] also prepared biodegradable gelatin microspheres using an aqueous solution of glutaraldehyde cross-linked gelatin to encapsulate hepatocyte growth factor (HGF) for its sustained release. The results revealed that the sustained release carrier improved liver cell function in rats with liver cirrhosis and showed promise as an effective therapy for liver fibrosis.

#### 2.1.2. Alginate and Its Derivatives

Alginate is a natural linear polysaccharide that can be extracted from the cell walls of seaweed, kelp, and other edible algae. Because of its low toxicity, high biocompatibility, and low price, alginate has been widely used in drug delivery and regenerative medicine [17].

Jeon et al. [18] found that delivery carriers based on photocrosslinked alginic acid and hyaluronic acid have controllable biodegradability [19] and can promote cell adhesion and provide sustained release of growth factors [20]. The researchers grafted heparin to alginate hydrogel and controlled the release of growth factors through heparin in the hydrogel. This hydrogel could provide sustained release of growth factors [transforming growth factor-*β* (TGF-*β*1), FGF-2, vascular endothelial growth factor (VEGF), and bone morphogenetic protein (BMP-2)] for up to three weeks, and no initial burst release was observed. Released growth factors could better promote the proliferation of human umbilical vein endothelial cells and alkaline phosphatase activity in osteoblasts. Liu et al. [21] found that alginate microspheres containing single or mixed growth factors (VEGF, FGF, and NGF) could efficiently release a variety of growth factors* in vitro* for more than four weeks. The three growth factors (VEGF, FGF, and NGF) encapsulated in alginate microspheres can generate synergies and better prolong survival of stem cells and promote myogenic differentiation of stem cells while enhancing peripheral nerve regeneration. The results showed that alginate microspheres containing the three growth factors and urine-derived stem cells produced significant effects in an animal model of incontinence. Further studies are needed to develop their potential use in incontinent patients.

#### 2.1.3. Chitosan and Its Derivatives

Chitosan is a product from deacetylation of chitin and is a natural polymer of sugar monomers. Because of its excellent blood compatibility, biological safety, and microbial degradability, it has been widely used in tissue engineering. Chitosan is a primary derivative of chitin. It is insoluble in water, but soluble in dilute acid, and can be absorbed by the body. Chitosan is a basic cationic polysaccharide polymer and has unique physical, chemical, and bioactive properties. Chitosan-based membranes, sponges, and microspheres encapsulating growth factors have been extensively studied and applied [22]. It has been reported that bFGF-containing chitosan can provide sustained release of bFGF and ensure the sustained release of bFGF over a long period of time [23]. Park et al. [24] designed a porous chondroitin sulfate-chitosan sponge that encapsulated platelet-derived growth factor (PDGF-BB), which provides controlled release of the growth factor by modulating the structure of porous scaffold to increase bone formation rate. Chondroitin sulfate was added to the chitosan solution, which was freeze-dried, cross-linked with tripolyphosphate, and freeze-dried again to yield a porous chondroitin sulfate-chitosan sponge. PDGF-BB solutions at different concentrations were added to the prepared sponge. The sponge was then left to stand at 4°C overnight and freeze-dried to yield the porous chondroitin sulfate-chitosan sponge containing 100, 200, and 400 ng of PDGF-BB, respectively. The chondroitin sulfate-chitosan sponge thus obtained had a pore size of 150–200 microns, providing porous structures needed for cell migration and bone growth. The release rate of PDGF-BB can be controlled by changing the amount of chondroitin sulfate added to the sponge and the initial loading dose of PDGF-BB. The results showed that the application of the chondroitin sulfate-chitosan sponge to provide sustained release of PDGF-BB at the wound site enhanced the adaptability and regenerative potential of osteoblasts.

Platelet lysate is an autologous source of growth factors and contains some bioactive agents capable of acting on bone regeneration. In the study by Santo et al. [25], chondroitin sulfate and chitosan were first prepared into nanoparticles, followed by the addition of platelet lysate; subsequently, polylactic acid foams were used to encapsulate platelet lysate-containing chitosan-chondroitin sulfate nanoparticles using supercritical fluid foaming technology to achieve controlled release of platelet lysate. The results showed that platelet lysate-containing nanoparticles could more quickly stimulate osteoblast differentiation and induce bone regeneration.

### 2.2. Synthetic Materials

Natural materials have been widely used as sustained release carriers for growth factors because of favorable biocompatibility, biodegradability, and low immunogenicity. However, they also suffer from some drawbacks, such as potential contamination in production and sterilization and poor mechanical properties. To overcome these problems, synthetic materials have emerged as an alternative for researchers. The synthetic materials allow greater flexibility in the control of their physical and chemical properties during production. Generally the monomer composition, reaction rate, and the molecular weight of composites can be controlled, which means that molecular weight, monomer species, and reaction rate can be manipulated to improve mechanical strength and change the rate of degradation.

#### 2.2.1. PEAD

Chu et al. [10] prepared PEAD from the polymerization of monomers arginine, aspartic acid, glycerol, and ethylene glycol.* In vitro* cell culture experiments showed that PEAD was not cytotoxic at a concentration of 1 mg/mL. Meanwhile, subcutaneous injection of 1 mg PEAD did not cause adverse reactions in rats. In addition, potential measurements showed that, like hyaluronic acid, PEAD had high affinity with polyanions such as DNA, which lays the foundation for PEAD to be used as a polymer for modifying protein drugs such as growth factors.

Heparin-binding EGF has been shown to effectively accelerate skin wound healing. Therefore, reducing the amount of growth factors through the use of heparin is very necessary for clinical efficacy. To achieve effective growth factor delivery, Johnson and Wang [26] designed a polymer-growth factor coacervate by using heparin-binding growth factor and attracting growth factor-containing heparin with PEAD through polyvalent charge [27].* In vivo* animal experiments showed that the coacervate provided sustained release of the growth factor and significantly accelerated wound healing in 17 days, significantly better than the control group, which used no growth factor, and the heparin-binding growth factor group. This result suggests that the polymer-heparin-growth factor delivery system can improve the biological activity of growth factors and hence accelerate skin wound healing.

#### 2.2.2. PLLA and Its Derivatives

PLLA membranes have widely been used in controlled drug release systems [11]. Park et al. [28] loaded PDGF-BB to porous PLLA membranes using the atmospheric drying phase inversion technique to provide controlled release of growth factors. PDGF-BB release can be controlled by altering the amount of bovine serum albumin added and the amount of initially loaded PDGF-BB. PLLA membranes encapsulating PDGF-BB significantly promoted new bone formation and completed bone remodeling two weeks after implantation in rats with skull defects. The results showed that PDGF-BB-incorporated PLLA membranes may enhance the guidance of potential tissue regeneration. Wang et al. [29] recently prepared a novel biomodified copolymer, poly(D,L-lactide)-7co-(1,3-trimethylene carbonate) (P (DLLA-co-TMC)), for controlling the release of vascular endothelial cell growth factor (VEGF). The results showed that the release of VEGF did not have a burst effect and that significant therapeutic effects were achieved.

#### 2.2.3. PLGA

PLGA has been widely used as the carrier for growth factors because of its favorable biocompatibility and biodegradability. The use of multiple emulsion technique (water/oil/water) can yield an encapsulation efficiency of up to 42% to 100% for growth factors [30]. The degradation rate of PLGA can be controlled by changing the amount of monomers lactic acid and glycolic acid, thereby controlling the rate of release of growth factors.

Spiller et al. [31] encapsulated IGF-1 in biodegradable PLGA microspheres using the emulsion method, which were then incorporated in polyvinyl alcohol (PVA) hydrogel.* In vitro* experiments showed that the release of IGF-1 from the hydrogel lasted for more than six weeks. IGF-1 is an important growth factor involved in the regeneration of cartilage. Its sustained release can enhance the formation of cartilage around the hydrogel and promote the integration of cartilage and the hydrogel. The results also demonstrated that the controlled release of growth factors can guide specific tissue regeneration and stem cell differentiation. Wang et al. [32] encapsulated PEGylated EGF in PLGA nanoparticles and erythropoietin (EPO) in PLGA nanoparticles via a poly(sebacic acid) coating to control the release of EGF and EPO. PEGylated EGF and EPO polymeric particles were dispersed in a hyaluronan methylcellulose (HAMC) hydrogel which spatially confines the particles and attenuates the inflammatory response of brain tissue. The results showed that, in a mouse model of stroke, sequential and sustained release of EGF and EPO from the microsphere system could better repair nerve tissue while causing no damage to other accompanying tissues as compared with intracerebroventricular infusion. This technique provides a minimally invasive and effective treatment modality for loss of healthy nerve cells and glial cells as a result of a number of central nervous system disorders such as Alzheimer's disease and spinal cord injury.

## 3. Inorganic Materials

It is well known that organic materials are widely used as sustained release carriers for growth factors, but extensive research efforts are also focused on inorganic materials used alone or in combination with organic materials, particularly in bone repair. Combining growth factors with inorganic materials can also achieve certain sustained release effects. More examples of inorganic materials include calcium phosphate and hydroxyapatite, and there are also various composites of organic and inorganic materials, such as porous hydroxyapatite/collagen (HAp/Col).

### 3.1. Calcium Phosphate

Calcium phosphate scaffolds (e.g., *β*-tricalcium phosphate [33]) offer favorable biocompatibility, excellent biological activity, and adjustable degradation rate [34, 35], making them an ideal material for bone repair. Calcium phosphate scaffolds used for bone repair often contain human primary cells (such as mesenchymal stem cells, bone cells, and endothelial cells) and growth factors. Growth factors can be encapsulated and embedded within the calcium phosphate scaffolds in a particular way. These factors can then be released into the microenvironment where bone grafting occurs to play a regulatory role by stimulating the expression of relevant genes.

Sun et al. [36] examined cell responses induced by the release of growth factors from a biodegradable porous calcium phosphate three-dimensional scaffold. They simulated and reconstructed a three-dimensional system for bone regeneration and assessed the effects of pore size and porosity on bone formation and angiogenesis. The results showed that, compared with pore size, porosity played a leading role in bone formation and angiogenesis and that pore size could adjust the rate of release of growth factors. The model developed by the institute can use specific scaffolds for sustained release of growth factors to predict bone regeneration.

### 3.2. Hydroxyapatite

Hydroxyapatite features excellent biocompatible and osteoconductive properties and easily combines with other materials [37]. It is an excellent carrier material and is widely used in sustained release of growth factors. It has a composition similar to inorganic bone matrix. Tsurushima et al. [38] coated hydroxyapatite ceramic buttons (HAP-CBs) with FGF-2 by precipitation in supersaturated calcium phosphate solution. HAP-CBs coated with high or low doses of FGF-2 were denoted by FGF-H or FGF-L. The release profile and biological activity of FGF-2 released from FGF-H and FGF-L were evaluated. The results showed that FGF-2 could still be detected at day 14 after* in vivo* release and the released growth factor still retained its biological activity. Bone formation is known to require a specific concentration of FGF-2, and this HAP-CB delivery system can release a sufficient amount of FGF-2 to induce bone formation.

The use of inorganic materials in association with various organic materials is more common than the use of inorganic materials alone. For example, although inorganic materials such as ceramics have potential benefits for bone replacement as high compressive strength, biodegradability, and osteoconductivity, they lack intrinsic mechanisms for controlled delivery. In this regard, the design of composite scaffolds is highly advantageous to control the release of growth factors. Fei et al.prepared a bone graft composite consisting of rhBMP-2 loaded PLGA microspheres and calcium phosphate cement achieving a controlled release for 28 days [39]. And Maehara et al. [40] impregnated porous hydroxyapatite/collagen (HAp/Col) with FGF-2 to repair large cartilage defects in rabbits. The scaffold was composed of hydroxyapatite nanocrystals and type I atelocollagen. The results showed that the FGF-2-incorporated porous hydroxyapatite/collagen effectively enhanced cartilage repair in rabbits. Letic-Gavrilovic et al. [41] also coupled collagen/hydroxyapatite (Col/HAp) to nerve growth factor *β* (NGF-*β*) for sustained release of the neurotrophin. The results of scanning electron microscopy and histological examination showed that this composite offers a variety of advantages in tissue engineering, such as excellent mechanical strength and biocompatibility, and is very suitable as a biomaterial for filling irregular maxillofacial defects.

## 4. Conclusions and Outlook

Growth factors are a class of versatile regulatory peptides secreted by a variety of cells and regulate important functions such as cell division, proliferation, and migration. Despite their widespread application in the clinic, major challenges remain with the use of growth factors, such as short half-life, susceptibility to inactivation, and rapid dilution and metabolism if used topically. In recent years, notable progress has been made in the research of materials for sustained release of growth factors. The use of natural polymer materials and modification of existing inorganic and organic polymer materials have emerged as an area of intense research for sustained release carriers. Studies have shown that sustained release composite systems can avoid burst release and ensure gradual and stable release of growth factors within certain concentrations. Moreover, it is possible to improve controlled and site-specific drug delivery and therefore enhance the bioavailability of growth factors by harnessing the properties of polymer materials, such as biocompatibility and tissue targeting. This represents a new direction in the research of carriers for sustained release of growth factors.

## Figures and Tables

**Table 1:** Lists of GFs and their main functions.

Type of GFs	Main functions	Reference
bFGF	Promote cell proliferation and regeneration	[42–44]

VEGF	Induce angiogenesis	[45–47]

TGF	Regulation of cell growth, differentiation, and immune function	[48–50]

NGF	An important regulator of neural survival	[51, 52]

IGF	Regulate the somatic growth in an endocrine manner	[53–55]

**Table 2:** New material and method for controlled release of varied GFs.

Types of GFs	Types of materials	Strategies for loading GFs	*In vitro*/*in vivo* effect	References
VEGF	PEG-heparin hydrogel	3D porous matrix through photocrosslinker along with the foaming process	Only 34% released over 13 days; more increase in angiogenesis *in vivo *	[56]

bFGF	PMMA-b-PMAETMA	Self-assembled core-shell of heparinized CS/c-PGA nanoparticles	Preserved heparin-bFGF biological activity *in vitro *	[57]

NGF	Iron oxide nanoparticles	Covalently conjugating the factor to iron oxide NP	Significantly promoted neurite outgrowth and increased the complexity of the neuronal branching trees *in vitro *	[58]

IGF-1	Collagen-GAG scaffold	Monitoring the amount of collagen and proteoglycan synthesized by chondrocytes seeded within the scaffolds	Provided an initial therapeutic burst release of IGF-1 which is beneficial in initiating ECM deposition and repair in the *in vitro* model	[59]

TGF-*β*3	PLCL scaffold	Supercritical CO_2_-HFIP cosolvent system	Long-term delivery of TGF-*β*3 prevented the hypertrophy of differentiated chondrocytes	[60]

VEGF and bFGF	Fibrin-based scaffold	Fibrin-based scaffold containing PLGA nanoparticles loaded with VEGF and bFGF	Complete reepithelialization, with enhanced granulation tissue formation, maturity, and collagen deposition	[61]
